# Supplementary material for: Drosophila SLC22 Orthologs Related to OATs, OCTs, and OCTNs Regulate Development and Responsiveness to Oxidative Stress
Source: Int J Mol Sci. 2020 Mar 15;21(6):2002. doi: 10.3390/ijms21062002 (PMC7139749; doi:10.3390/ijms21062002)
Supplement: Supplementary file 1 [file ijms-21-02002-s001.pdf]

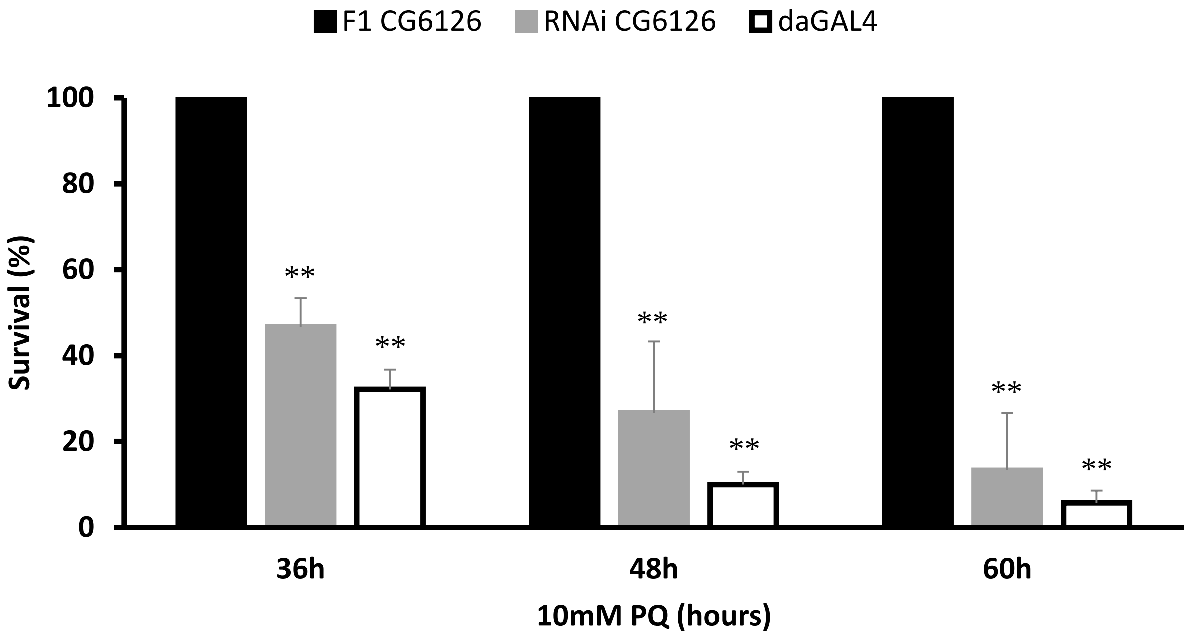

Supplementary Figure 1: *CG6126* RNAi knockdown shows resistance to oxidative stress. Parent lines: BDSC Stock 56038, red eye *da⁻GAL4*. Error bars represent standard error (S.E.), F1 v RNAi p=0.0012051 and F1 v daGAL4 p=0.0010053 at 36h. F1 v RNAi p=0.0010053 and F1 v daGAL4 p=0.0010053 at 48h. F1 v RNAi p=0.0010053 and F1 v daGAL4 p=0.0010053 at 60h. **p<0.01.

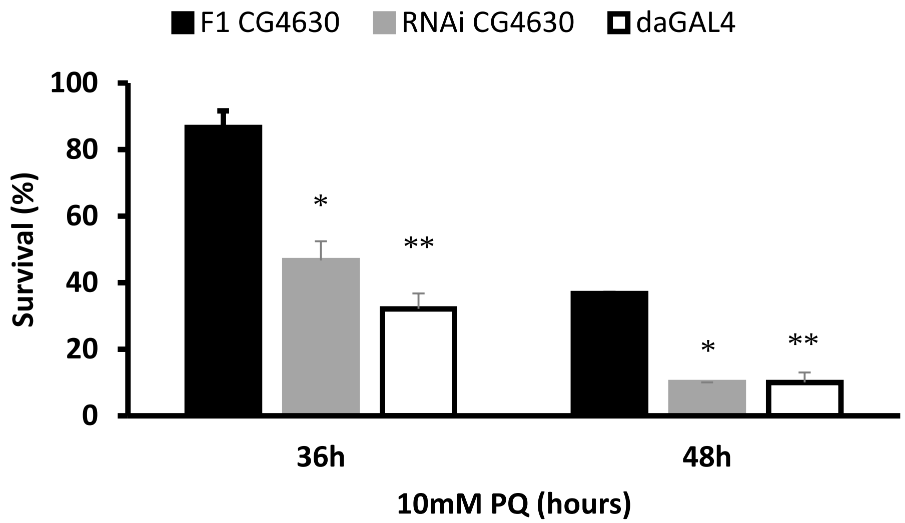

Supplementary Figure 2: *CG4630* RNAi knockdown shows resistance to oxidative stress. Parent lines: BDSC Stock 61249, red eye *da⁻GAL4*. Error bars represent standard error (S.E.), F1 v RNAi p=0.021328; F1 v daGAL4 p=0.0010053 at 36h. F1 v RNAi p=0.0295573; F1 v daGAL4 p=0.0055523 at 48h. * p<0.05, **p<0.01.

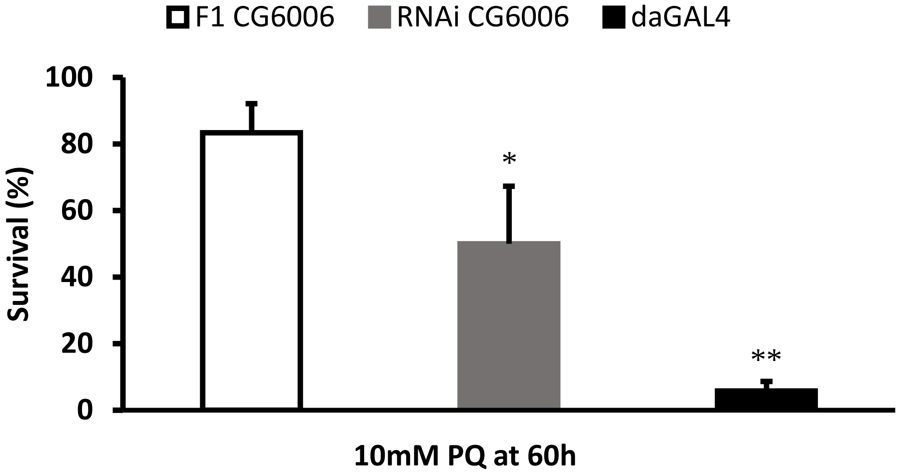

Supplementary Figure 3: *CG6006* RNAi knockdown shows resistance to oxidative stress. Parent lines: BDSC Stock 55282, red eye *da⁻GAL4*. Error bars represent standard error (S.E.), *indicates time point with statistically significant increased percent survival of F1 when compared to both parent lines. F1 v RNAi p=0.0362421; F1 v daGAL4 p=0.0010053 at 60h. * p<0.05, **p<0.01.

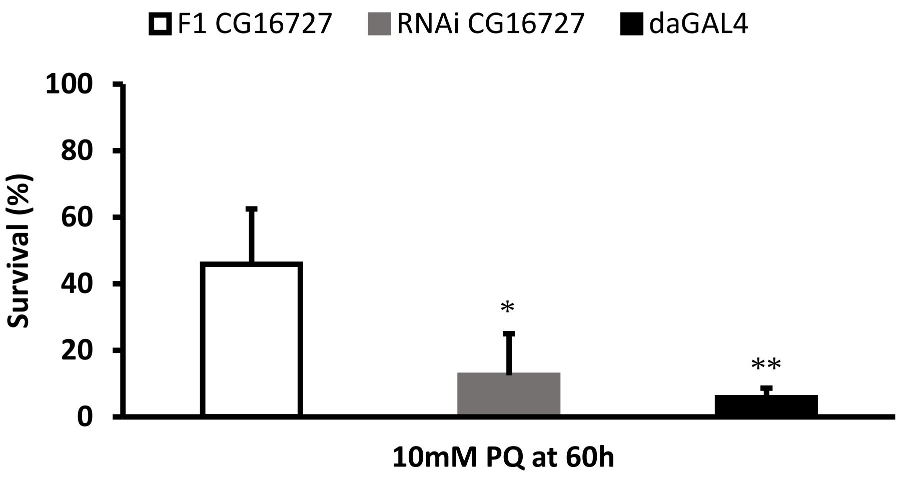

Supplementary Figure 4: ***CG16727*** **RNAi knockdowns show resistance to oxidative stress**. Parent lines: BDSC Stock 57434, red eye *da*$^{-}$*GAL4*. Error bars represent standard error (S.E.), *indicates time point with statistically significant increased percent survival of F1 when compared to both parent lines. F1 v RNAi p=0.0453956; F1 v daGAL4 p=0.00233 at 60h. * p<0.05, **p<0.01.
